# 298. Successful Treatment of Persistent Severe Acute Respiratory Syndrome Coronavirus 2 Infection in an Immunocompromised Patient

**DOI:** 10.1093/ofid/ofac492.376

**Published:** 2022-12-15

**Authors:** Victoria Overbeck, Jacquelyn Turcinovic, Xueting Qiu, Scott Seitz, John Connor, Beau Schaeffer, William P Hanage, Scott R Curry, Krutika Kuppalli

**Affiliations:** Boston University School of Public Health, Brookline, Massachusetts; Boston University, Boston, Massachusetts; Harvard T.H. Chan School of Public Health, Boston, Massachusetts; Boston University, Boston, Massachusetts; Boston University, Boston, Massachusetts; Harvard T.H. Chan School of Public Health, Boston, Massachusetts; Harvard T. H. Chan School of Public Health, Boston, Massachusetts; Medical University of South Carolina, Charleston, South Carolina; World Health Organization, Geneva, Geneve, Switzerland

## Abstract

**Background:**

The COVID-19 pandemic has been marked by long-term persistence of SARS-CoV-2 in immunocompromised patients receiving B-cell-depleting therapies, with many individuals experiencing fatal COVID-19.

**Methods:**

We report an individual treated with rituximab who survived persistent COVID-19 over 9 months. SARS-CoV-2 positive RNA samples were sequenced using targeted amplicon NGS sequencing with backup sequencing on a nanopore platform. The resulting sequences were analyzed for genomic variance over time at the consensus and sub-consensus level.

**Results:**

An individual with rheumatoid arthritis (RA) treated with azathioprine and rituximab (last dose in May 2020) was diagnosed with COVID-19 in July 2020 and admitted with pneumonia. After initial incomplete recovery, the patient had persistent infection through March 2021 and received both remdesivir and convalescent plasma (January 2021). The patient received three doses of mRNA vaccine (Pfizer BioNTech) in December 2020, April 2021, and November 2021, but was seronegative for nucleocapsid IgG in both January 2021 and March 2021; positive spike IgG developed by September 2021 (512 AU/ml) and December 2021 (621 AU/ml) (Figure 1). The patient recovered with new oxygen dependence (2-3L) and manages RA off B-cell depletion; they required an extended corticosteroid taper to manage organizing pneumonia and treatment for several opportunistic infections.

Viral sequencing over the course of illness indicated a persistent infection with a lineage B.1.585.3 virus that accumulated 14 mutations throughout the infection. Two mutations (S494P, S D737Y) are associated with therapy resistance and are similar to those found in other immunocompromised individuals with persistent COVID-19. Additional mutations were of unknown consequence.

**Figure 1:**
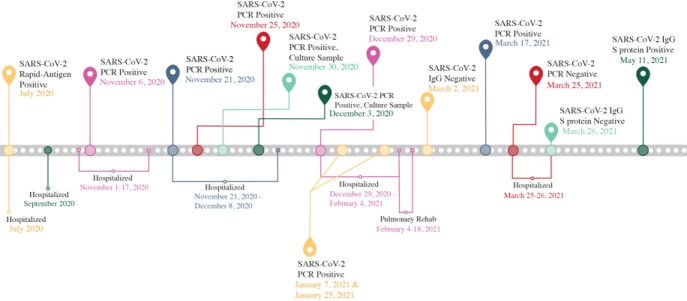
Timeline of Immunocompromised Patient’s Clinical Course of COVID-19

**Conclusion:**

SARS-CoV-2 was able to establish persistent infection and accumulated mutations associated with therapeutic resistance; repeated vaccination was associated with successful resolution following repeated vaccination after stopping rituximab. Cessation of B-cell-depleting therapy was likely the critical factor in the patient’s recovery, but repeated vaccination was associated with a delayed seroconversion in this patient with reversibly immunosuppression.

**Disclosures:**

**William P. Hanage, PhD**, Biobot Analytics Inc: Advisor/Consultant|Merck Vaccines: Advisor/Consultant.

